# Exploring How Pain Leads to Productivity Loss in Primary Care Consulters for Osteoarthritis: A Prospective Cohort Study

**DOI:** 10.1371/journal.pone.0120042

**Published:** 2015-04-07

**Authors:** Ross Wilkie, Elaine M. Hay, Peter Croft, Glenn Pransky

**Affiliations:** 1 Arthritis Research UK Primary Care Centre, Primary Care Sciences, Keele University, Keele, Staffordshire, ST5 5BG, United Kingdom; 2 Center for Disability Research, Liberty Mutual Research Institute, 71 Frankland Rd., Hopkinton, Massachusetts, 01748, United States of America; University of Calgary, CANADA

## Abstract

**Objective:**

Osteoarthritis pain has become a leading cause of decreased productivity and work disability in older workers, a major concern in primary care. How osteoarthritis pain leads to decreased productivity at work is unclear; the aim of this study was to elucidate causal mechanisms and thus identify potential opportunities for intervention.

**Methods:**

Population-based prospective cohort study of primary care consulters with osteoarthritis. Path analysis was used to test proposed mechanisms by examining the association between pain at baseline, and onset of work productivity loss at three years for mediation by physical limitation, depression, poor sleep and poor coping mechanisms.

**Results:**

High pain intensity was associated with onset of work productivity loss (Adjusted Odds Ratio 2.5; 95%CI 1.3, 4.8). About half of the effect of pain on work productivity was a direct effect, and half was mediated by the impact of pain on physical function. Depression, poor sleep quality and poor coping did not mediate the association between high pain intensity and onset of work productivity loss.

**Conclusions:**

As pain is a major cause of work productivity loss, results suggest that decreasing pain should be a major focus. However, successfully improving function may have an indirect effect by decreasing the impact of pain on work productivity, especially important as significant pain reduction is often difficult to achieve. Although depression, sleep problems, and coping strategies may be directly related to work productivity loss, addressing these issues may not have much effect on the significant impact of pain on work productivity.

## Introduction

Osteoarthritis is the most common joint condition in adults and globally is the fastest increasing major heath condition [1–2]. It is a common reason for primary care consultation (one out of every twenty consultations in adults aged between 45 and 65 is primarily for osteoarthritis), and is also a common comorbidity in persons seen in primary care for other reasons [3]. This condition begins to emerge as a major cause of functional limitations and work disability from age 50 years onwards, and with aging of the population, has become a leading and rapidly growing cause of decreased productivity and premature exit from employment [4]. Although one in four workers with osteoarthritis leave the work place prior to normal retirement age, the majority remain in employment [5]. However many individuals with osteoarthritis who remain employed have health-related difficulty or reduced productivity (presenteeism) on the job [6,7]; on average they experience a third less productivity on the job compared to their same-age coworkers [2]. More of them will be expected to delay retirement and continue employment due to shrinking retirement resources. As a result, identifying effective approaches to better sustain productive employment in OA patients has become an international priority.

**Fig 1:**
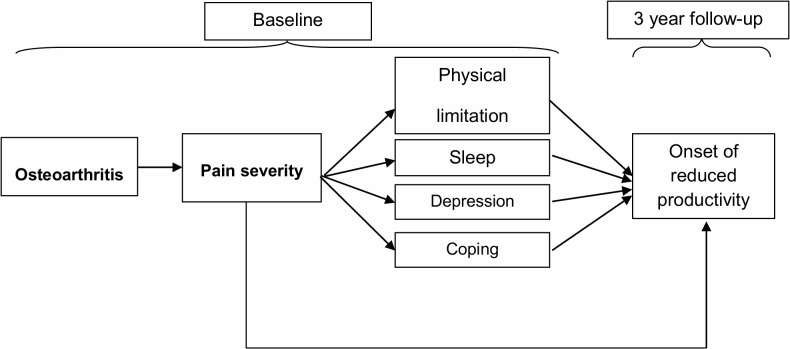
Hypothesised pathways between pain and the onset of reduced productivity among primary care consulters with OA.

Exactly how osteoarthritis impacts work ability is not well understood [8]. Most studies conclude that either joint pain or poor function are a major driver of work productivity loss in osteoarthritis, but cross-sectional design and lack of examination of causal pathways limits a more in-depth exploration. One possibility is that joint pain primarily affects work productivity through a direct effect, independent of comorbidities or other causal mechanisms. Alternatively, the impact of joint pain might be primarily through an indirect pathway, where joint pain causes physical limitations, depression, poor coping and poor sleep quality which in turn lead to work productivity loss [9–13] (Fig. 1). Understanding the mechanism of how osteoarthritis affects the ability to continue work productivitycan help to focus future management and preventative strategies.

The availability of a longitudinal observational cohort study from a large, representative primary care cohort provided a unique opportunity to evaluate alternative causal pathways through path analysis techniques. The aim of this study was to provide an in-depth examination of how pain leads to the onset of work productivity loss and identify potential new intervention opportunities. Such an examination is timely, as studies have demonstrated that pain treatment by itself does not often yield significant improvements in work productivity or prevent subsequent work disability in other chronic noninflammatory musculoskeletal conditions [14, 15]. Our hypothesis was that joint pain in osteoarthritis patients primarily causes presenteeism through a direct effect on work productivity, rather than an indirect causal pathway (where the primary causes of presenteeism are mediating factors such as decreased physical function, depression, and poor sleep quality, each in part caused by joint pain).

## Method

### Study population

The North Staffordshire Osteoarthritis project (NorStOP) is a population-based prospective cohort study. The NorStOP sampling frame comprised all individuals aged 50 years and over who were registered to receive care from one of six general practices in North Staffordshire, England, United Kingdom (UK). In 2008, records of adults who gave their written consent for medical record review were evaluated to identify persons who received a diagnosis of osteoarthritis during a primary care consultation 2000 and 2008. They were mailed questionnaires in 2005 (baseline) and 2008 (three-year follow-up); reminders were sent at two and four weeks after the initial mailing. The North Staffordshire Local Research Ethics Committee approved this study.

Analyses for this paper included those who (i) consulted for osteoarthritis from 2000 to 2008 (the study period of Norstop)), (ii) were of working age (less than 65 years old) and in employment at the three year follow-up (2008) and (iii) completed the items on work productivity at baseline (2005) and three-year follow-up (2008).

### Identification of osteoarthritis

General practitioners in the study used the Read system to code all reasons for clinical encounters in primary care consultations [16]. The Read codes cross-map to ICD9/ICD-10 (for diseases). Morbidity data (i.e. symptoms and diseases) in this system are grouped into 19 Read chapters. Data on these diagnostic groups were aggregated starting in 2000, continuing through the time of the follow-up questionnaire in 2008. Individuals were defined as having osteoarthritis if they had at least one consultation during this period primarily for osteoarthritis based on Read codes (N05 category) for primary care consultations [16]. As osteoarthritis is a long-standing, gradually progressive chronic condition, it was assumed that a clinician-established diagnosis at any point during the study period implied that osteoarthritis was likely present at least to some degree during the entire period of observation.

### Onset of work productivity loss

Work productivity was measured using a single item from the Medical Outcomes Study Short Form-36 [17] at both survey time points. Participants were asked “During the past 4 weeks, have you accomplished less than you would like in your work or other regular daily activities as a result of your physical health?”; yes/no. For this analysis “yes” was used to define loss in work productivity. To measure onset of loss in work productivity at follow-up, persons with loss in work productivity at baseline were excluded and onset was defined as movement from no loss (i.e. response of no) at baseline to loss of work productivity at 3 year follow-up.

### Pain status

Pain status, all mediators and confounders were measured at baseline. Pain intensity was measured using the Short Form-36 item “How much bodily pain have you had during the past 4 weeks?” [17] and classified as high (moderate, severe, very severe) or low (none, very mild, mild).

### Potential Mediators

Physical function was measured using the physical functioning scale of the Medical Outcomes Study Short Form-36; score range: 0–100, higher scores indicating better function [17]. Sleep quality was measured using a single item from the Jenkins Sleep Questionnaire [18]. The question asks about recent problems with non-restorative sleep, which predicts poor outcomes in older adults [15]; During the past four weeks did you wake up after your usual amount of sleep feeling tired and worn out? (not at all/on some nights/on most nights). For this analysis “on most nights” was used to define poor sleep quality [19]. Levels of depression were measured using the Hospital Anxiety and Depression scale (HAD) [20]. It consists of 7 items scored on a Likert scale of 0–3 and which gave a total score of 0–21. Poor coping mechanisms were measured using the Coping Strategies Questionnaire [21]. Each of the seven items capture a scale of the Coping Strategies Questionnaire; diverting attention, reinterpreting pain sensations, catastrophizing, ignoring sensations, praying and hoping, coping self-statements and increased behavioural activities. Each item is scored 0–6 on a numerical rating scale with verbal anchors (never do that, always do that) to give a total score 0–42.

### Potential confounders

Possible confounders included demographic factors (age, gender), socio-economic status (represented by occupational class: professional/managerial, semi-routine, routine); educational attainment (further education, or not) and comorbidity. Read codes at the second hierarchical level or above were used to identify any consultations for each comorbidity category, during the period between 2002 and 2005. The number of different comorbidities consulted for were then summed to give a total score ranging from 0 to 19.

### Statistical analysis

First, distribution and rates of potential mediators and confounders were compared by whether or not onset of work productivity loss had occurred, with differences tested for significance using Chi-square or Kruskall Wallis tests where appropriate. Logistic regression was then used to test the relationship between pain intensity at baseline and the onset of work productivity loss three years later in an unadjusted model, then adjusting subsequently for age, gender, occupational class, education and comorbidity.

Path analysis (an extended form of multiple regression which tests whether dependent variables are part of a causal pathway for the occurrence of an outcome [22]) was used to test the proposed mechanisms by examining for mediation of the association between pain intensity at baseline and the onset of work productivity loss at 3 years by baseline levels of physical limitation, depression, poor quality sleep and coping at baseline. A series of models were built to estimate (i) the total effect of pain at baseline on onset of work productivity loss at three years (without adjustment for other mediators), (ii) the direct effect (i.e. the effect of pain on onset work productivity loss adjusting for pathway variables), and (iii) the indirect effect (i.e. the reduction in the total effect of pain on onset of work productivity loss minus the direct effect). The indirect effect indicates the “amount” of mediation and the extent to which each putative mediator explains the link between baseline pain and onset of work productivity loss [23]. The Karlson-Holm-Breen (khb) method of decomposition was adopted to separate the total effect in a logistic model into direct (pain) and indirect (physical limitation, depression, poor sleep quality and poor coping mechanisms) effects [23]]. The proportion of mediation is calculated by dividing the indirect effect by the total effect [24], and this can be interpreted as the proportion of pain effects that might be explained by physical limitation, depression, poor sleep quality and poor coping mechanisms. The first model examines the total effect of pain on onset of loss of work productivity. Physical limitation, depression, poor sleep quality and coping were then added separately to estimate the extent that each variable mediates the association between pain and onset. Putative confounders were added to each model. Results were reported as standardized beta coefficients.

## Results

**Fig 2:**
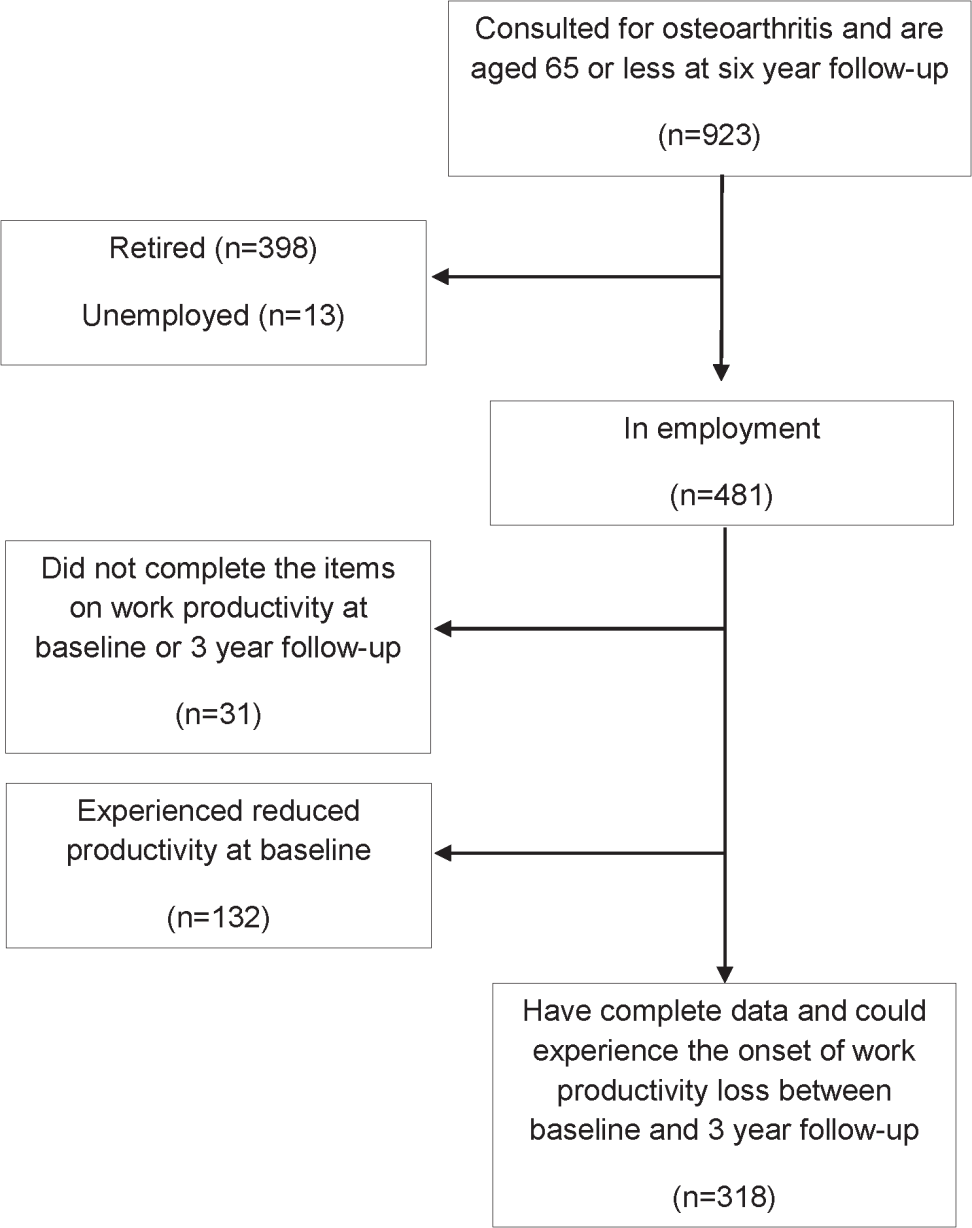
Flow diagram of participants.

Over the study period there were 923 adults who had consulted for osteoarthritis and were of working age at 3 year follow-up. Of this group 398 had retired before state retirement age, 13 were unemployed, 31 were homemakers, leaving 481 who were in employment and thus eligible for the study. Of these, 31 did not have complete data, leaving complete data for 450 participants. Compared to those subjects with incomplete data (n = 31) those included in the analysis (n = 450) were more likely to be younger (60.4 years cf 56.2 years; p<0.001) but no more likely to be female (p = 0.19), have better physical (p = 0.84) or mental health (p = 0.54), have gone onto further education (p = 0.27) or have manual occupations (p = 0.63). At baseline 132 (29.3%) were already experiencing reduced work productivity, leaving 318 for the analysis (Fig. 2).

### Participant characteristics

At baseline, mean age overall was 56.2 (Standard deviation: 2.2) years, 52.8% were women and 77.9% had a high school education only (Table 1). Of the 318 participants in the final cohort, 91 (28.6%) participants reported a high intensity pain and 53 (16.7%) reported the onset of work productivity loss. Onset of work productivity loss was more frequent in those with high pain intensity (p<0.01), poor sleep quality (p = 0.01) and with lower levels of physical function (p<0.01). The rate of onset of work productivity loss was unrelated to gender (p = 1.00), education (p = 0.80), manual occupations (p = 0.80), depression (p = 0.27), number of comorbidities (0.93) or coping mechanisms (p = 0.69).

**Table 1:** Subject characteristics at baseline overall and by pain extent.

	Overall	No onset	Onset	P value
	(n = 318)	(n = 265)	(n = 53)	
**Age**				
Mean (standard deviation) years	56.2 (2.2)	56.6 (2.2)	56.2 (2.2)	0.12
**Gender**				
No. (%) Female	168 (52.8)	140 (52.8)	28 (52.8)	1.00
**Pain**				
No. (%) High intensity	91 (28.6)	68 (25.7)	23 (40.4)	<0.01
**Education**				
No. (%) No further education	244 (77.9)	202 (77.7)	42 (79.3)	0.80
**Occupational class**				
No. (%) Manual occupation	134 (42.7)	111 (42.2)	23 (44.2)	<0.001
**Comorbidity**				
Median (IQR)	2 (1–4)	2 (1–4)	2 (1–4)	0.93
**Physical function**				
Median (IQR)	85 (65–95)	90 (80–95)	85 (65–90)	<0.001
**Depression**				
No. (%) Possible/probable cases	16 (5.0)	11 (4.1)	5 (9.4)	0.27
**Poor sleep quality**				
No. (%)	42 (13.6)	29 (11.3)	3 (25.3)	0.01
**Coping mechanism**				
Mean (standard deviation)	1.83 (0.99)	1.82 (1.01)	1.89 (0.90)	0.69

### Association between pain and the onset of loss in work productivity

High pain intensity at baseline was significantly associated with the onset of work productivity loss three years later (Odds ratio 2.2 (1.2, 4.1). This association remained unchanged when adjusted for age, gender, educational attainment, occupational class and comorbidity (adjusted OR 2.5 (95%CI 1.3, 4.8). Age (0.9; 0.8, 1.0), gender (1.0; 0.5, 1.8), education (1.0; 0.5, 2.2), occupational class (1.2; 0.6, 2.2) and comorbidity (1.4; 0.8, 2.6) were not associated with onset of loss in work productivity.

### Association between pain and onset of work productivity loss at 3 years, via physical limitation, depression, poor sleep quality and poor coping mechanisms

Physical limitation mediated the association between pain intensity and the onset of work productivity loss but depression, poor sleep quality and poor coping mechanisms did not (Table 2). The standardized beta coefficient (β) for the total effect of pain on the onset of work productivity loss was 0.44 (95% confidence interval: 0.11, 0.76). After inclusion of physical limitation as a mediator, the standardized beta coefficient for the direct effect of pain on work productivity was 0.22 (-0.12, 0.56) and the indirect effect was 0.22 (0.09, 0.34). Depression, poor sleep quality and poor coping mechanisms did not mediate the association between high pain intensity and onset of work productivity loss. When added separately, the direct effect was 0.42 (0.11, 0.74), 0.40 (0.07, 0.72) and 0.46 (0.11, 0.81) respectively and the indirect effect was 0.02 (-0.02, 0.05), 0.07 (-0.01, 0.14) and 0.00 (-0.07, 0.06).

**Table 2:** The pathway from pain at baseline to the onset loss of work productivity at three year follow-up via physical limitation, depression poor sleep quality and poor coping mechanisms.

	Physical Limitation	Depression	Poor sleep quality	Poor coping mechanisms
	Effect size† Coefficient (SE)	Effect size† Coefficient (SE)	Effect size† Coefficient (SE)	Effect size† Coefficient (SE)
Total effect	0.44 (0.17)	0.44 (0.16)	0.46 (0.16)	0.46 (0.17)
Direct effect	0.22 (0.17)	0.42 (0.16)	0.40 (0.17)	0.46 (0.18)
Indirect effect	0.22 (0.07)	0.02 (0.02)	0.07 (0.04)	0.00 (0.03)

**†** adjusted for age, gender, educational attainment, occupational class and comorbidity

## Discussion

In a population of consulters for osteoarthritis, high pain intensity was strongly associated with a subsequent onset of work productivity loss. This relationship was mediated by physical limitation, but not by depression, poor sleep quality or poor coping mechanisms. Thus, high pain intensity had both a direct (independent) and an indirect effect on the onset of work productivity loss. Socio-demographic factors (age, gender, educational attainment, occupational class) and comorbidity were also not associated with onset of work productivity loss.

Although the impact of pain and reduced physical function on work outcomes have been previously reported [25, 26] the longitudinal approach and mediation analysis enable us to better explore the mechanism of pain causing work productivity loss. As physical limitation explained 50% of the total effect of pain on the onset of work productivity loss, this provides an explanation of why pain levels may correlate poorly with changes in function at home and at work in chronic musculoskeletal conditions [27,28]. Depression, sleep quality and coping have been implicated in work disability and work productivity loss in several health conditions [29, 30], but these results suggest that the effects are primarily direct, and not indirectly caused by higher pain levels.

These results imply that especially in patients with higher pain levels, therapeutic efforts to target physical functional improvement may not only have a direct effect on work productivity outcomes, but also significantly reduce the impact of pain on work productivity, even without a substantial reduction in pain levels. Observational studies demonstrate that many persons in the general populations with substantial levels of musculoskeletal pain maintain a fairly high level of function and participation, often independent of seeking health care [28]. This finding is also consistent with the approach of multidisciplinary functional restoration programmes to achieving positive work outcomes [31]. These programs incorporate a multidisciplinary approach that targets both direct pain effects, as well as enhancing function as a separate and desirable outcome, achieving work outcomes that are consistently better than in unimodal, pain-centred treatments [32]. Recent reports on the risks and lack of significant functional impact of opioid treatment for chronic MSD also support the importance of considering a broader therapeutic paradigm instead of a narrow focus on pain reduction [33]. Depression, insomnia and coping have been found to be associated with work productivity and targeting these issues may improve work outcomes but not though an impact on pain—related effects on work. When pain appears to be the major barrier to participation in work and other activities, additional treatment focused directly on improving function may be more effective than targeting depression, sleep or coping.

Ageing populations and extensions to working life means that there will be more older workers with osteoarthritis in future years. The focus on work productivity and presenteeism is important because most adults of working age with osteoarthritis remain in the work place. Osteoarthritis is associated with significant impact on work productivity, but not long-term work absenteeism in this cohort [5]. In this study at baseline almost one in three reported reduced work productivity and a further 16% developed this problem over a three year period. Future studies could focus on whether addressing these mediating effects through function-oriented interventions actually do lead to a decrease in the pain-work productivity relationship, especially where pain levels are higher and not easily reduced.

The study has a number of strengths. The longitudinal design enables prospective identification of factors associated with the onset of productivity loss in a clinically relevant primary care population. The sample is representative of primary care consulters with physician diagnosed osteoarthritis, relevant to primary care practices. Other studies have been limited to patients from rheumatology practices or rehabilitation clinics, a less representative sample of osteoarthritis patients (e.g. [34]). The available data covered a number of important areas in relation to the onset of work productivity loss and specifically factors that may influence pain reporting in older people.

There are limitations to this study. Data on most variables was from self-report, but validated instruments were used to measure all variables. Although the outcome variable measured accomplishing less in regular activities in addition to work, prior studies and interviews with patients suggest that for those in employment, work activities were the focus for their responses [35]. The question about limitations was not specific to pain, and onset of work productivity loss could be due to other conditions besides OA; these issues would bias results towards the null. Information on radiographic findings or clinical information on the extent of OA was unavailable, but the intention of the study was to describe a typical, heterogeneous group of patients with OA as seen in primary care practice. Consultation for OA for some participants will have been identified after baseline data was collected. However as OA is a long standing, gradually progressive chronic condition, it can be assumed that OA will have been present prior to consultation and when data was collected. Measuring the predictors at one time point three years before the outcome may not reflect changes in these factors during follow-up. Evaluation of cases with missing data indicated there may be some bias due to differences in age, but not due to gender, socio-economic and health status, but it is less likely that attrition resulted in biased results [36]. A simple count of comorbidity was included in which all conditions were weighted equally. This approach may be insufficient to fully explain the relationship of co-occurring comorbidities, as it does not account for the severity of individual conditions or interaction among co-occurring conditions. A number of factors that may mediate or moderate the association between pain intensity and work productivity loss were not available, for example job control, accommodations, and support at the workplace from supervisors and coworkers [37].

We excluded persons who transitioned from employment to early retirement for several reasons, even though some of these persons may have left employment because of osteoarthritis-related work productivity decreases. We do not have information on why they retired early, and thus the reasons could include non-osteoarthritis conditions, or adequate finances that enabled early retirement (e.g. spouse income, pension, savings). An earlier study with this cohort found that work loss in this age group was not significantly related to osteoarthritis [5]. Also, in persons who are not working, the outcome question is more likely to be related to current non-work activities, not prior employment.

In conclusion, the findings from this prospective cohort study of primary care consulters for osteoarthritis identify function as an important mediator of the impact of pain on subsequent decreases in work productivity. These results reinforce the value of targeting pain intensity, and suggest that, especially with high pain intensity, addressing physical function limitations will reduce the subsequent onset of work productivity loss. This offers a promising alternative approach to current pain-centred therapeutic options that are having limited success. The increasing number of older workers with osteoarthritis and its chronic nature means that targeting these factors may have a substantial impact on maintaining work productivity in older workers [38]. Medical approaches to managing the pain are important and targeting pain is going to make some contribution to preventing or reducing work productivity loss. Physiotherapy and exercise classes that address the limitations to physical function may also be useful. Future studies could focus on intervention studies which target pain and physical function and their effect on work productivity.
